# Perioperative Care of a Six-Year-Old Child With Atlanto-Occipital Dislocation: A Focus on Techniques of Airway Management

**DOI:** 10.14740/jmc5289

**Published:** 2026-03-27

**Authors:** Marwan Hillis, Edison E. Villalobos, Christopher Lancaster, Tariq Wani, Joseph D. Tobias

**Affiliations:** a Department of Anesthesiology and Pain Medicine, Nationwide Children’s Hospital, Columbus, OH, USA; b Department of Anesthesiology and Pain Medicine, The Ohio State University College of Medicine, Columbus, OH, USA

**Keywords:** Atlanto-occipital dislocation, Spinal cord injury, Trauma, Cervical spine

## Abstract

Atlanto-occipital dislocation (AOD), once regarded as a rare and almost universally fatal injury identified primarily at autopsy, is now recognized with increased frequency in surviving trauma patients. Improved diagnostic imaging, coupled with advances across the entire continuum of trauma care, has resulted in the potential survivability of this devastating injury. Despite the high mortality associated with this devastating injury, survival with successful outcome is feasible. Airway management in patients with AOD must balance minimizing cervical spine movement to prevent exacerbation of neurologic injury, continued resuscitation and maintenance of cerebral blood flow, while securing the airway to ensure effective oxygenation and ventilation. We present a 6-year-old female patient who sustained significant polytrauma, including AOD, and who, after cervical spine fusion and instrumentation, subsequently presented for elective surgical repair of an associated brachial plexus injury. The mechanisms underlying AOD are described, key anatomical considerations of the cervical spine are reviewed, and the perioperative management of patients with this complex injury is discussed with a focus on the various techniques used for airway management during various surgical and radiologic procedures.

## Introduction

Atlanto-occipital dislocation (AOD), once regarded as a rare and almost universally fatal injury identified primarily at autopsy, is now being recognized with increasing frequency in surviving trauma patients [1, 2]. This shift is the result of substantial advances in diagnostic imaging, as well as progressive improvements across the entire continuum of trauma care, including prehospital field management, transport systems, and advanced trauma life support in specialized medical centers. Although AOD was first described in 1908, the first documented case of survival was not reported until nearly four decades later [3, 4]. However, the devastating nature of this injury is illustrated by high mortality rates; for example, in a retrospective review, Hosalkar et al reported death in 50% of patients (eight of 16 patients) with AOD on arrival to the emergency department, while an additional three died intraoperatively due to extensive intracranial injuries [5]. Among patients with cervical spinal injuries who survive to hospital admission, the reported incidence of AOD is approximately 1%, whereas postmortem studies dating from 1970s reported AOD in 6–8% of patients involved in fatal accidents, with children showing a significantly higher incidence than adults [1, 6].

We present a 6-year-old female patient who sustained significant polytrauma, including AOD, and who, after cervical spine fusion and instrumentation subsequently presented for elective surgical repair of an associated brachial plexus injury. The mechanisms underlying AOD are described, key anatomical considerations of the cervical spine are reviewed, and the perioperative management of patients with AOD is discussed.

## Case Report

Review of this case and presentation in this format followed the guidelines of the Institutional Review Board of Nationwide Children’s Hospital. Written consent was obtained for the use of deidentified patient information for publication.

The patient was a 6-year-old girl, weighing 22.1 kg with a history of polytrauma following a high-velocity motor vehicle collision. She was an unrestrained back-seat passenger and was ejected approximately 80–100 feet out of the car. She sustained a severe traumatic brain injury, a large left forehead degloving laceration, cervical spine trauma, bilateral pulmonary contusions, a right hepatic lobe laceration, a traumatic perianal laceration, and multiple fractures involving the thoracic vertebral bodies (T2, T6, T7, T8), sacral segments (S1–S3), and multiple extremities. Family history and past medical history were unremarkable. On arrival to our institution via emergency medical services, she presented with a Glasgow Coma Scale (GCS) of 6. Intravenous (IV) ketamine (2 mg/kg) and rocuronium (1 mg/kg) were administered, after which the cervical collar was briefly removed and manual in-line cervical stabilization was maintained to facilitate videolaryngoscopy (C-MAC, Macintosh blade size 3) (Table 1). A Cormack-Lehane grade 1 view was noted, and endotracheal intubation was achieved on the first attempt with a 5.0-mm cuffed endotracheal tube (ETT) assisted by a stylet. The cervical collar was replaced, and IV volume resuscitation was initiated, followed by adjunctive medications including mannitol, levetiracetam, and a fentanyl infusion. Ongoing hemodynamic instability prompted urgent transfer to the pediatric intensive care unit (PICU) for further stabilization. Initial computed tomography (CT) scan imaging demonstrated no calvarial fracture or intracranial hemorrhage but raised concern of significant spinal trauma including AOD. Given her low GCS and the need for further diagnostic evaluation, she was transported to the operating room (OR) for emergent placement of a right frontal external ventricular drain, repair of scalp and perineal laceration followed by magnetic resonance imaging (MRI) acquisition under general anesthesia. She arrived in the OR with the previously placed ETT still in place and tolerated the procedure without intraoperative adverse effects. Intraoperative MRI was obtained with full cervical spine precautions in close coordination with the neurosurgical team. Following completion of imaging, the patient’s trachea remained intubated, and she was transported back to the PICU for ongoing postoperative care. MRI revealed extensive craniocervical junction instability confirming the diagnosis of AOD, a T2 Chance-type fracture, and multilevel thoracic and sacral fractures. On postoperative day (POD) 2, she returned with the ETT in place from her initial procedure for occiput to C3 fusion and sublaminar T1 to T3 fixation to prevent neurologic deterioration and restore structural integrity. General anesthesia was administered, and the patient remained hemodynamically stable throughout the procedure. A halo cranial fixation device and vest were placed intraoperatively, and she was transported back to the PICU. Over the ensuing 5–7 days, there was progressive improvement in her neurologic status. She was awake, following commands, and her neurologic examination was nonfocal. On hospital day 12, the patient’s trachea was successfully extubated, and respiratory support was transitioned to nasal cannula with supplemental airway clearance therapies. Two days later, she returned to the OR for examination under anesthesia and laparoscopic colostomy due to progressive breakdown of her perineal wound. Anesthesia was induced with propofol (3 mg/kg), and neuromuscular blockade was provided by rocuronium (1 mg/kg). Airway management was challenging due to halo immobilization and limited jaw mobility. Bag-valve-mask ventilation improved following placement of an oral airway. Eventually, orotracheal intubation was achieved on the second attempt using a two-provider combined technique of videolaryngoscopy (C-MAC, Macintosh blade size 2), flexible fiberoptic bronchoscopic-guided endotracheal intubation, and cricoid pressure, which yielded a Cormack–Lehane grade IIb view. A 5.0-mm cuffed ETT, which was threaded over the bronchoscope, was advanced under direct visualization using the flexible bronchoscope as a rigid guide and visualization with the C-MAC video laryngoscope. Correct placement was confirmed by bronchoscopy, capnography, chest rise, and bilateral breath sounds. The procedure was successfully completed, and the patient’s trachea was extubated in the OR before transferring to the PICU.

**Table 1:** Outline of Anesthetic Care and Airway Management Techniques

Timeline	Airway management
Initial ER presentation	Ketamine (2 mg/kg) and rocuronium (1 mg/kg) were administered. The cervical collar was removed, and manual in-line cervical stabilization was maintained to facilitate VL (C-MAC, Macintosh blade size 3). Cormack–Lehane grade 1 view was noted, and a 5.0-mm cuffed ETT with stylet was placed on the first attempt.
OR for ventriculostomy and then MR	Patient arrived in the OR with the previously placed ETT still in place and tolerated the procedure without intraoperative adverse effects.
Hospital day 2–3 for posterior cervical fusion	Returned with ETT in place from the initial placement in the emergency department. General anesthesia was administered, and the patient remained hemodynamically stable throughout the procedure. A halo cranial fixation device and vest were placed intraoperatively, and she was transported back to the PICU.
Hospital day 12	Trachea successfully extubated in the PICU.
Hospital day 14–15	Returned to the OR for examination under anesthesia and laparoscopic colostomy due to progressive breakdown of her perineal wound. Anesthesia was induced with propofol (3 mg/kg) and neuromuscular blockade provided by rocuronium (1 mg/kg). Airway management was challenging with halo immobilization and limited jaw mobility. Bag-valve-mask ventilation improved with placement of an oral airway. Orotracheal intubation was achieved on the second attempt using a two-provider combined technique of VL and flexible fiberoptic bronchoscopic-guided endotracheal intubation, and cricoid pressure, which yielded a Cormack–Lehane grade IIb view. A 5.0-mm cuffed ETT, threaded over the bronchoscope, was advanced under direct visualization with VL. The patient’s trachea was extubated in the OR before transferring to the PICU.
Four subsequent procedures after discharge from the hospital: procedure 1 (examination under anesthesia of perineum)	Halo device in place, and airway management was uncomplicated and achieved using an air-Q intubating laryngeal mask (LMA) size 2.5.
Procedure 2: reconstruction of the perineum	An initial attempt with VL was unsuccessful: a Cormack–Lehane grade IIb view and esophageal intubation. Second attempt triggered laryngospasm, which was treated with succinylcholine. Successful intubation was achieved using a combined technique with air-Q LMA and flexible fiberoptic bronchoscopy. The air-Q LMA was used as a conduit to allow successful advancement of a 5.0-mm cuffed ETT, which was threaded over the bronchoscope, and passed into the trachea.
Procedure 3: anorectal exam and colostomy closure	Halo immobilization had been discontinued a month earlier. Initial airway assessment with direct laryngoscopy revealed redundant oropharyngeal tissue and inability to extend the neck. Ramped fashion, head held by one provider above the bed with minimal flexion while cricoid pressure was applied. Orotracheal intubation was then attempted using VL, Cormack–Lehane grade IIb view and placement of a 5.0-mm cuffed ETT on the first attempt.
Procedure 4: brachial plexus exploration and reconstruction	Anesthesia was induced by the inhalation of sevoflurane in oxygen. A peripheral IV cannula was placed after achieving adequate depth of anesthesia, followed by an IV bolus of propofol (4 mg/kg) and fentanyl (2.5 µg/kg). Bag-mask ventilation was confirmed to be easy, and the patient’s trachea was intubated using VL (Glidescope, LowPro blade size 2.5), assisted by a bougie.

ER: emergency room; ETT: endotracheal tube; VL: videolaryngoscopy; PICU: pediatric intensive care unit; OR: operating room; MR: magnetic resonance; IV: intravenous.

**Figure 1:**
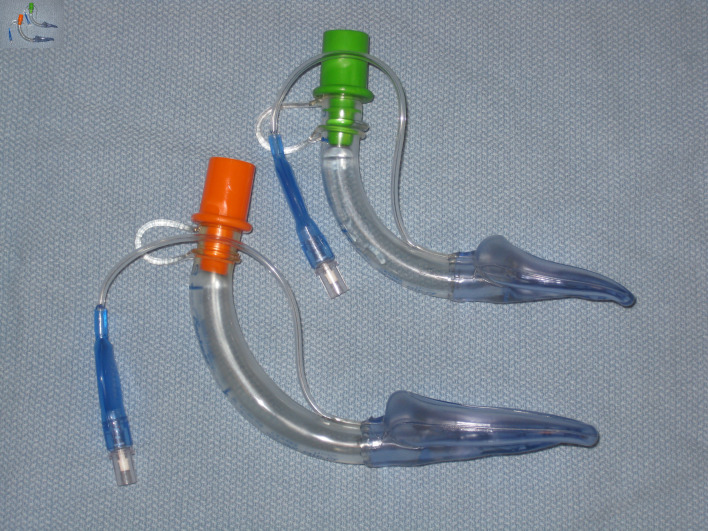
Photograph of standard air-Q laryngeal mask airways used for airway management. The wider lumen and shorter stem with removable 15-mm adaptor allows for easier passage of an endotracheal tube during fiberoptic guided endotracheal intubation as used in our patient.

**Figure 2:**
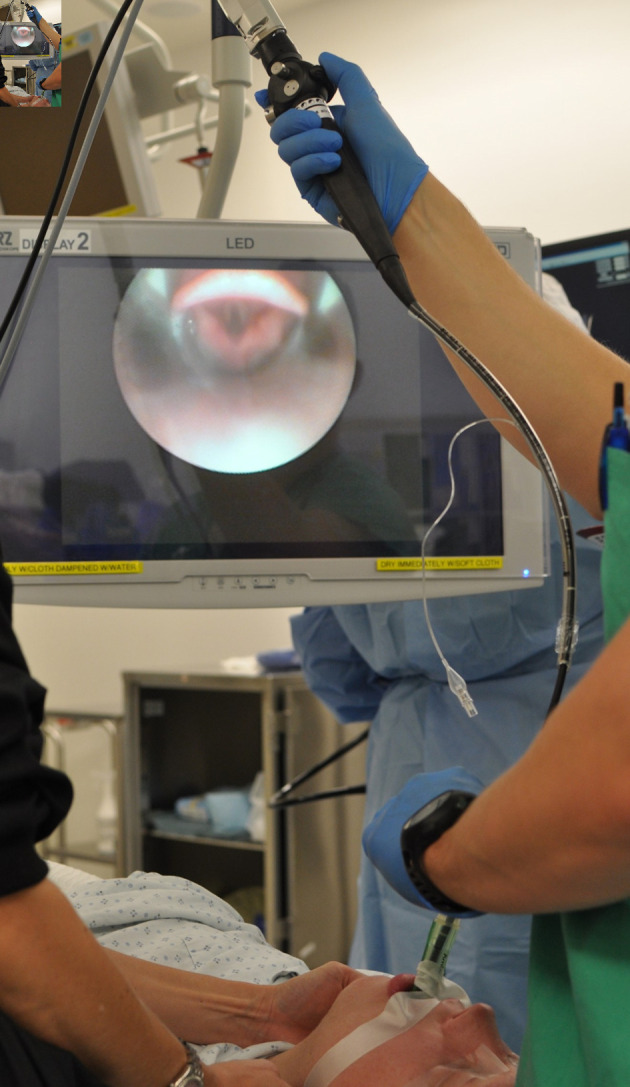
Demonstration of technique of fiberoptic bronchoscopy use for direct visualization and guidance of endotracheal intubation through an LMA. LMA: laryngeal mask airway.

Overall, the patient experienced a prolonged and complex hospital course requiring multidisciplinary care focused on maximizing functional independence, pain management, cognitive recovery, wound care, and behavioral support. The total hospital length of stay was 47 days. After discharge, she underwent four additional scheduled procedures. The first of these procedures was an examination under anesthesia of perineum and anus of approximately 40 min of anesthetic care. The patient still had a halo device in place, and airway management was uncomplicated, achieved using an air-Q intubating laryngeal mask airway (LMA), size 2.5. The second procedure was a reconstruction of the perineum, and given the anticipated duration of the surgery and the need for neuromuscular block, endotracheal intubation was chosen. With the patient’s head on the sniffing position, an initial attempt using videolaryngoscopy (C-MAC, Macintosh blade size 2) provided a Cormack–Lehane grade IIb view but resulted in esophageal intubation. A subsequent attempt was complicated with laryngospasm, which was treated with succinylcholine. Definitive airway control was achieved using a combined technique with air-Q LMA and flexible fiberoptic bronchoscopy. The air-Q LMA was used as a conduit to allow successful advancement of a 5.0-mm cuffed ETT, which was threaded over the bronchoscope, and passed into the trachea (Figs. 1, 2). A Cook soft-tipped extra-firm airway exchange catheter (11 Fr, 100 cm) was placed through the ETT, followed by removal of the air-Q LMA, while the exchange catheter and ETT remained *in situ* in order to maintain continuous tracheal access and limit the risk of ETT dislodgement. Endotracheal intubation was confirmed by bronchoscopy and capnography, and anesthesia was maintained for approximately 2 h.

The third procedure was an anorectal examination under anesthesia and colostomy closure. Halo immobilization had been discontinued a month earlier. Initial airway assessment with direct laryngoscopy revealed redundant oropharyngeal tissue and inability to extend the neck. With the patient positioned in a ramped fashion, the head was held by one provider above the bed with minimal flexion while cricoid pressure was applied. Orotracheal intubation was then attempted using videolaryngoscopy (C-MAC, Macintosh blade size 2), yielding a Cormack–Lehane grade IIb view and allowing successful placement of a 5.0-mm cuffed ETT on the first attempt. Tracheal intubation was atraumatic, and the patient remained hemodynamically stable throughout approximately 4 h of anesthetic care.

**Figure 3:**
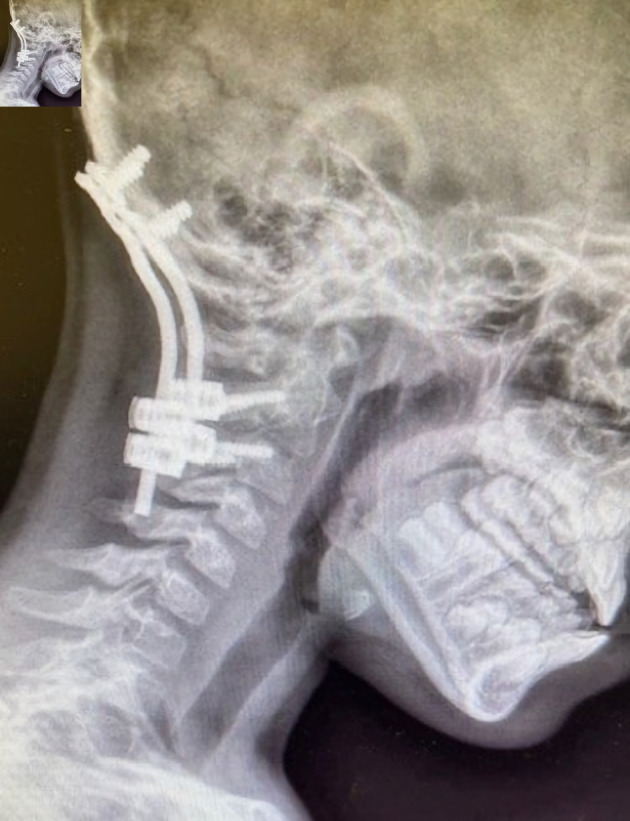
Preoperative radiographs demonstrating cervical spine instrumentation for stabilization following atlanto-occipital dislocation.

The fourth procedure was a left brachial plexus exploration and reconstruction with nerve grafting and direct intraoperative nerve stimulation to assess muscle responses. The complexity of this case relied on preserving neuromuscular function for reliable nerve stimulation while ensuring cervical spine and airway protection. Perioperative vital signs showed a temperature of 37.2 °C (99 °F), pulse 109 beats/min, blood pressure 102/77 mm Hg, and oxygen saturation of 100% on room air. On physical examination, there was limited neck flexion and extension, as well as the past history of airway concerns and the previous cervical fusion and instrumentation (Fig. 3). Cardiac and respiratory examinations were otherwise unremarkable. The patient was held *nil per os* (NPO) for 8 h before the surgery. Routine American Society of Anesthesiologists monitors were applied, and anesthesia was induced by the inhalation of sevoflurane (8%) in oxygen. A peripheral IV cannula was placed after achieving adequate depth of anesthesia, followed by an IV bolus of propofol (4 mg/kg) and fentanyl (2.5 µg/kg). Bag-mask ventilation was confirmed to be easy, and the patient’s trachea was intubated 2 min later using videolaryngoscopy (Glidescope, LowPro blade size 2.5), yielding a Cormack–Lehane grade I view and allowing successful placement of a 5.0-mm cuffed ETT assisted by a bougie. Placement was confirmed with capnography, chest rise, and bilateral breath sounds. To facilitate muscle responses during intraoperative nerve stimulation, neuromuscular blocking agents (NMBAs) were avoided. Maintenance anesthesia consisted of sevoflurane (2%), remifentanil infusion (0.1–0.3 µg/kg/min), and supplemental IV boluses of fentanyl (1 µg/kg), hydromorphone (0.015 mg/kg), dexamethasone (0.3 mg/kg), and acetaminophen (15 mg/kg). Ondansetron (0.1 mg/kg) was administered for nausea prophylaxis, and cefazolin (50 mg/kg every 3 h) was given to prevent surgical site infections. A total volume of 627 mL of lactated Ringer’s solution was administered during 8 h of anesthetic care. The patient’s trachea was extubated in the OR when fully awake, and she was transferred to the post-anesthesia care unit (PACU) in stable hemodynamic condition. The patient was discharged home on POD 1. A wide range of cervical spine precautions were applied during each anesthetic encounter, and further details regarding our observations and management are described below.

## Discussion

Securing the airway in trauma patients with a potentially unstable cervical spine, or following surgical stabilization, can be a complex and challenging task, especially in urgent or emergent scenarios after traumatic injury. Airway management must balance minimizing cervical spine movement to prevent exacerbation of neurologic injury, continued resuscitation and maintenance of cerebral blood flow, while securing the airway to ensure effective oxygenation and ventilation [7–11]. In patients with suspected or confirmed cervical spine trauma or instability, airway management may be required because of respiratory or hemodynamic compromise or in the setting of elective or emergent procedures requiring general anesthesia.

The cervical spine is a complex structure stretching from the skull base (occiput) down to the first thoracic vertebral body and is composed of seven cervical vertebrae (C1–C7) [12–14]. It is functionally and structurally divided into two distinct regions: the specialized occipito-atlanto-axial complex (C0–C2) and the sub-axial spine (C3–C7). The upper cervical spine comprises three unique bony elements tailored for head movement: the occiput, the ring-like atlas (C1), and the axis (C2). The atlas (C1) is distinguished by its lack of a vertebral body or spinous process. The axis (C2) is characterized by the prominent odontoid process (or dens), which projects superiorly from the vertebral body into the C1 ring, serving as the essential pivot point for rotational movement. The lower, sub-axial segment (C3–C7) consists of five vertebrae that are anatomically similar to the thoracic and lumbar segments, with the crucial distinction being the presence of bilateral transverse foramina, which contain the ascending vertebral arteries that provide the vascular supply of the posterior brain.

The cervical spine permits movement across three axes (flexion/extension, rotation, and lateral bending); however, airway manipulation primarily utilizes flexion and extension. Cervical spine motion is not uniformly distributed across its segments. The occiput-C1 (C0–C1) joint allows for significant extension (15°–20°), but minimal flexion (approximately 5°). The C1–C2 joint, while paramount for axial rotation, contributes approximately 10° to both flexion and extension. Approximately 65° of further flexion/extension is gained from the lower cervical spine, with most of it being attributable to the caudal segments.

The lateral aspects of the atlas articulate with the occiput through paired semilunar condyles in a relatively unstable format. Stability is provided by strong surrounding ligaments that connect the occiput not only to the atlas but also to the axis, which explains the relatively common associated distraction injury of C1–2. The more immature the spine is, the flatter the joints are. This, in conjunction with underdeveloped and lax ligaments and these flatter joints, results in an unstable fulcrum during high-energy trauma, a vulnerability that is further aggravated by the disproportionately larger heads of children compared with their bodies. The most frequently injured ligaments in AOD, the tectorial membrane and the alar ligaments, are also the most important stabilizing ligaments in the cranio-cervical junction. Injury to these two ligaments is the most detrimental to the patient, but additional ligaments are often injured during trauma, as evidenced during surgery or autopsies. These factors lead to a great predisposition to AOD injury in children who are less than 8 years of age.

The clinical presentation of AOD is highly variable, ranging from the absence of discernible neurological deficits to severe neurologic impairment secondary to brainstem, spinal cord, or lower cranial nerve injury, and may progress to quadriplegia, ventilator dependence, and death [15, 16]. Given the high-energy mechanisms typically involved, associated injuries including and beyond the craniospinal axis are common. Clinical manifestations of AOD may include respiratory irregularities, cardiac arrhythmias, and motor deficits such as quadriparesis, cruciate paralysis, or crossed hemiplegia/hemiparesis, depending on the level of pyramidal tract involvement. Multiple lower cranial nerve palsies are frequently observed, most often involving the abducens (cranial nerve VI), followed by the hypoglossal (cranial nerve XII). Cranial nerve dysfunction is the result of distraction forces causing either direct axonal injury or damage to the corresponding brainstem nuclei. Cervicomedullary junction injury results from a combination of tissue disruption from primary injury and secondary injury from inadequate or delayed immobilization, compression by displaced bony structures or epidural hematoma, and ischemia due to vertebral artery compromise. Given the proximity to their course with the vertebral foramina, vertebral artery injury may occur, necessitating a high index of suspicion for associated vascular injury, which may require additional diagnostic imaging such as MRI, computed tomographic angiography or formal catheter angiography. Spinal cord injury (SCI) is frequently associated with AOD, with a reported incidence ranging from 54% up to 100% [17–19].

Although various grading systems have been suggested for classification of AOD, the first system, proposed by Traynelis et al in 1986, is still the most commonly used [20]. It is based on the relationship between the occiput and the atlas including type I, anterior displacement of the occiput over the atlas; type II, longitudinal distraction; and type III, posterior displacement of the occiput over the atlas. Although it is widely used, it has been suggested that this classification has limited utility because of the inherent instability of the spine with AOD, and the high mobility of the occiput relative to the atlas may allow all three types of movement to be present in the same patient. Treatment options include halo immobilization with or without spinal fusion and instrumentation depending on the severity of the injury and the extent of the ligamentous/vertebral injury and instability.

As noted in our patient, the associated cervical spine injury, the immobilization devices used, and the duration since the traumatic event can significantly impact airway management and the techniques used to secure endotracheal intubation. Regardless of the clinical scenario, successful outcomes in patients with traumatic brain injury, including AOD, require immediate resuscitation and successful airway management [7–9]. Our patient demonstrates the various airway techniques which may be used depending on the clinical scenario along the continuum from the initial traumatic event through the hospital course and recovery. Airway management techniques are influenced by the preoperative assessment of the airway using standard assessment tools which is impacted by stabilization devices such as a cervical collar or halo, the specific procedure (surgical or radiologic) and its requirements including the need for the administration of NMBAs and endotracheal intubation, as well as the current clinical state of the patient (acute hemodynamic and respiratory state related to the traumatic event and comorbid conditions). In all scenarios, this requires preparation with availability and familiarity with the perioperative tools for airway management including standard laryngoscopes, oral/nasal airways, fiberoptic bronchoscopes, indirect videolaryngoscopy, laryngeal mask airways, airway catheters/bougies, and other devices commonly used in the management of the potentially difficult airway [21–25]. In our patient, the initial airway management with endotracheal intubation following the initial traumatic event adhered to standard trauma practices, with attention to stabilization of the cervical spine in a patient with a rigid cervical collar in place. The trachea was intubated using a standard rapid sequence intubation protocol with manual in-line stabilization of the cervical spine and indirect videolaryngoscopy. Subsequent airway encounters were made challenging by the presence of rigid immobilization devices (halo) and limited jaw mobility. In these encounters, airway management required the combination of direct visualization of the airway using indirect videolaryngoscopy and ETT placement by direct fiberoptic guidance with placement of the ETT over the bronchoscope and guidance into the airway. In one encounter, this was accomplished using an LMA as a conduit. Alternatively for less invasive procedures, where endotracheal intubation and/or the use of NMBAs was not necessary, anesthetic care was successfully provided with the airway managed by placement of an LMA. Regardless of the technique used, successful use of these devices for airway management was facilitated by a through preoperative assessment and development of an airway plan and the ready availability of the needed equipment, contained in our difficult airway cart.

### Learning points

Survival following traumatic injury with AOD, previously regarded as a lethal injury, is now being recognized with increased frequency. Identification of this devastating injury may be feasible as a result of advances in diagnostic imaging with the potential for survival, as illustrated by our patient, due to improvements in the continuum of trauma care. Effective techniques for airway management in these scenarios are key in a successful outcome. Airway management must balance minimizing cervical spine movement to prevent exacerbation of neurologic injury, continued resuscitation and maintenance of cerebral blood flow, while securing the airway to ensure effective oxygenation and ventilation. Various airway management techniques may be used depending on the clinical scenario, ranging from the initial traumatic event through the hospital course and recovery. These techniques are influenced by the preoperative assessment of the airway using standard assessment tools which is impacted by stabilization devices such as a cervical collar or halo, the specific procedure (surgical or radiologic) and its requirements including the need for the administration of NMBAs and endotracheal intubation, as well as the current clinical state of the patient (acute hemodynamic and respiratory state related to the traumatic event and comorbid conditions). In the scenarios encountered in our patient, this required standard laryngoscopes, oral/nasal airways, fiberoptic bronchoscopes, indirect videolaryngoscopy, laryngeal mask airways, airway catheters/bougies, and other devices commonly used in the management of the potentially difficult airway. Additional information regarding airway management in this challenging clinical scenario is provided elsewhere [9, 26, 27]. Regardless of the technique used, successful use of these devices for airway management was facilitated by a thorough preoperative assessment and development of an airway plan and the ready availability of the needed equipment, contained in our difficult airway cart.

## Data Availability

The data supporting the findings of this study are available from the corresponding author upon reasonable request.
